# Retrospective Evaluation of Infants Aged 1 to 60 Days with Residual Cerebrospinal Fluid (CSF) Tested Using the FilmArray Meningitis/Encephalitis (ME) Panel

**DOI:** 10.1128/JCM.00277-18

**Published:** 2018-06-25

**Authors:** Anne J. Blaschke, Kristen M. Holmberg, Judy A. Daly, Amy L. Leber, Jennifer Dien Bard, Ernest K. Korgenski, Kevin M. Bourzac, Kristen J. Kanack

**Affiliations:** a University of Utah School of Medicine, Department of Pediatrics, Division of Pediatric Infectious Diseases, Salt Lake City, Utah, USA; b BioFire Diagnostics, LLC, Salt Lake City, Utah, USA; c Primary Children's Hospital, Salt Lake City, Utah, USA; d Nationwide Children's Hospital, Columbus, Oklahoma, USA; e Children's Hospital of Los Angeles, Los Angeles, California, USA; f Pediatric Clinical Program, Intermountain Healthcare, Salt Lake City, Utah, USA; Carter BloodCare and Baylor University Medical Center

**Keywords:** FilmArray, PCR, febrile infant, meningitis, molecular diagnostics, sepsis

## Abstract

In pediatric practice it is common for infants under 2 months of age to undergo evaluation for sepsis when they are ill, often including lumbar puncture to assess for central nervous system (CNS) infection. The FilmArray Meningitis/Encephalitis (ME) panel is a newly approved test for rapid identification of CNS pathogens. Our objective was to study the epidemiology of CNS infection in young infants and the potential impact of rapid multiplex PCR on their care. A performance evaluation of the FilmArray ME panel was conducted from February 2014 to September 2014 at 11 sites. FilmArray ME panel results were compared to reference standards but not shared with providers. In our study, medical records for infants (aged 1 to 60 days) enrolled at three sites were reviewed for clinical, laboratory, and outcome data. A total of 145 infants were reviewed. The median age was 25 days. Most of the infants were hospitalized (134/145 [92%]) and received antibiotics (123/145 [85%]), and almost half (71/145 [49%]) received acyclovir. One infant had a bacterial pathogen, likely false positive, identified by the FilmArray ME panel. Thirty-six infants (25%) had a viral pathogen detected, including 21 enteroviruses. All infants with enteroviral meningitis detected by the FilmArray ME panel and conventional PCR were hospitalized, but 20% were discharged in less than 24 h when conventional PCR results became available. The FilmArray ME panel may play a role in the evaluation of young infants for CNS infection. Results may be used to guide management, possibly resulting in a decreased length of stay and less antimicrobial exposure for infants with low-risk viral infection detected.

## INTRODUCTION

Infants in the first 2 months of life are at high risk for invasive bacterial infection, and it is the standard of care for them to undergo an evaluation for sepsis when they have fever or other concerning signs, such as lethargy or poor feeding (1). As part of this evaluation, many infants have a lumbar puncture (LP) performed to assess the possibility of central nervous system (CNS) infection. Most infants undergoing LP are hospitalized for empirical antimicrobial therapy while cultures and other pathogen-based tests are pending (1).

Bacterial meningitis and herpes simplex virus (HSV) encephalitis are severe infections that require prompt treatment with parenteral therapy (2, 3). Other viral encephalitides are often self-limiting, and care is supportive (1). Detection of certain viral pathogens, such as enterovirus (EV), in young infants evaluated for fever has been associated with a reduced likelihood of concomitant bacterial infection (4).

The introduction of molecular testing for viral illness into the care process for evaluating young infants for sepsis has led to a number of changes in management (5–7). Although infants used to be hospitalized and treated empirically with antibiotics for up to 72 h awaiting results of sterile site cultures (1, 8), many infants are now discharged in <24 h if they are clinically well and found to have a viral illness, and some are managed expectantly, without antibiotics (9, 10). More-rapid molecular testing with larger pathogen panels could lead to further improvements in management. A recent study in children, including a specific subset of infants with cerebrospinal fluid (CSF) tested for EV or HSV, suggested the potential for clinical impact of a rapid multiplex panel, including more-rapid diagnosis of CNS infection and optimization of antimicrobial therapy (11).

We performed a retrospective evaluation of clinical and outcome data for 145 infants 1 to 60 days old enrolled in the performance evaluation for the FilmArray (BioFire Diagnostics, LLC, Salt Lake City, UT) Meningitis/Encephalitis (ME) panel (12) at three sites. The FilmArray ME panel detects and identifies 14 pathogens that can cause meningitis and/or encephalitis, including 7 viruses, 6 bacteria, and 1 yeast (12). Our objectives were to understand the epidemiology of CNS infection in young infants undergoing diagnostic LP as determined by a combination of molecular and conventional diagnostics and to consider the possible impact of rapid pathogen-based multiplex testing for CSF on the management of these infants.

## MATERIALS AND METHODS

This study was approved by the Institutional Review Boards of the University of Utah and Primary Children's Hospital (Salt Lake City, UT), Children's Hospital Los Angeles (Los Angeles, CA), and Nationwide Children's Hospital (Columbus, OH).

### Patient selection, FilmArray testing, and clinical record review.

A prospectively enrolled performance study for the FilmArray ME panel was conducted between February and September of 2014 at 11 U.S. sites (12). The total enrollment was 1,560 subjects (adults and children) for whom a clinician had ordered a bacterial culture on CSF obtained by LP. A waiver of informed consent was obtained from the IRB at each site for testing of deidentified, remnant CSF specimens. A confidential enrollment log was maintained by a third party at each site in order to collect limited, deidentified information from the subject's medical record. Specimens were tested with the FilmArray ME panel, and the results were compared to standard-of-care (SOC) testing performed on the same specimen per clinician's orders at each site. SOC tests included bacterial culture, as well as independent molecular methods such as EV and HSV PCRs performed at each site (institution-specific laboratory-developed tests). The FilmArray ME panel results were coded such that they could not be reported back to the clinician or the subject and were not used to influence patient care. Data from this study and others were used to support regulatory applications for U.S. Food and Drug Administration and Conformité Européene (CE) marking in 2015 (12). Analytes on the FilmArray ME panel include six bacteria (Escherichia coli K1, Haemophilus influenzae, Listeria monocytogenes, Neisseria meningitidis, Streptococcus agalactiae, and Streptococcus pneumoniae), seven viruses (cytomegalovirus [CMV], EV, HSV-1, HSV-2, human herpesvirus 6 [HHV-6], human parechovirus [HPeV], and varicella-zoster virus [VZV]), and one yeast group (*Cryptococcus neoformans/C. gattii*).

For the present study, medical records for infants aged 1 to 60 days enrolled in the clinical performance study at 3 of the 11 sites were retrospectively interrogated for demographic, clinical, and outcome data. Clinical and outcome data included laboratory data, clinical presentation on admission, length of hospital stay (LOS), administered therapies and discharge diagnoses.

## RESULTS

PCR results and clinical data were available for review for 145 infants 1 to 60 days old. Demographics, clinical characteristics and results of the FilmArray ME panel and conventional testing are shown in Table 1. The median age of the children was 25 days (range, 2 to 59 days), and 62 (43%) of them were older than 28 days. Most (119/145; 82%) had fever on presentation; about half (77/145; 53%) had symptoms suggestive of meningitis, and almost all (134/145; 92%) were admitted to the hospital. Seventeen infants (12%) were treated in the intensive care unit (ICU). A total of 37 infants (26%) had pathogens identified by the FilmArray ME panel, and 21 (14%) had pathogens identified by conventional methods.

**TABLE 1:** Demographics and pathogens detected in 145 infants 1 to 60 days old

Parameter	Data
Demographic characteristics	
No. female (%)	55 (38)
Median age in days (range)	25 (2–59)
No. of infants older than 28 days (%)	62 (43)
No. (%) of subjects presenting with:	
Fever	119 (82)
Meningitis symptoms^*a*^	77 (53)
Appearance of sepsis^*b*^	2 (1)
No. (%) admitted to hospital	134 (92)
No. (%) treated in ICU	17 (12)
No. of pathogens detected in CSF as determined by FilmArray ME/conventional methods	
Escherichia coli K1	0/0
Haemophilus influenzae	0/0
Listeria monocytogenes	0/0
Neisseria meningitidis	0/0
Streptococcus agalactiae	0/0
Streptococcus pneumoniae	1/0
Cytomegalovirus (CMV)	1/0
Enterovirus (EV)	20^*c*^/17
Human herpesvirus 6 (HHV-6)	4/0
Herpes simplex virus 1 (HSV-1)	0/0
Herpes simplex virus 2 (HSV-2)	0/0
Human parechovirus (HPeV)	11^*c*^/4
Varicella-zoster virus (VZV)	0/0
*Cryptococcus neoformans/C. gattii*	1/0
Other	0/0^*d*^
Total pathogens	38/21

a Lethargy, irritable, bulging fontanelle, seizure.

b Low blood pressure, fluid resuscitation, pressor support, “meets sepsis criteria” in notes.

c One infant had both EV and HPeV detected by FilmArray ME for 37 infants with 38 detections.

d One Gram stain-positive (Gram-negative rods), culture-negative CSF.

Table 2 shows detailed clinical characteristics, laboratory data, and outcomes by pathogen detected. One infant had a bacterial pathogen (Streptococcus pneumoniae) identified by the FilmArray ME panel, and one had a fungal pathogen (Cryptococcus *neoformans/C. gattii*) identified. Neither child had abnormal CSF studies or positive conventional testing, and neither was diagnosed or treated for CNS infection. Secondary testing during the parent study could not confirm these detections, suggesting that these were both false-positive findings (12).

**TABLE 2:** Infant characteristics by type of pathogen detected using the CSF FilmArray panel or standard testing

Parameter	Value for pathogenic^*a*^:						
	Bacterium (S. pneumoniae, *n* = 1)	Virus				Yeast (Cryptococcus, *n* = 1)	None (*n* = 107)
		EV (*n* = 21)	HPeV (*n* = 11)	CMV (*n* = 1)	HHV-6 (*n* = 4)		
Demographics							
Median age in days (range)	12 (NA)	26 (3–54)	30 (7–52)	33 (NA)	45 (20–54)	10 (NA)	23 (2–59)
% female	0	48	64	100	50	100	32
No. (%) older than 29 days	0	9 (43)	7 (64)	1 (100)	3 (75)	0	43 (40)
No. (%) with a chronic medical condition	0	6 (29)	0	1 (100)	0	0	38 (36)
No. of pathogens detected by:							
FilmArray ME	1	20	11	1	4	1	108
Standard CSF test	0	17	4	NT	NT	NT	118
CSF parameters, no. (%) or median (range)							
CSF pleocytosis^*c*^	0	13 (62)	1 (9)	0	0	0	17 (16)
WBC count	3 (NA)	29 (0–1,779)	2 (0–20)	5 (NA)	6 (2–11)	7 (NA)	4 (0–70)
RBC count	560 (NA)	78 (0–58,130)	2 (0–21,000)	347 (NA)	64 (0–442)	1 (NA)	4 (0–103,300)
% neutrophils	21 (NA)	13 (0–92)	0 (0–17)	2 (NA)	1 (0–3)	1 (NA)	1 (0–67)
Protein	71 (NA)	79 (19–525)	44 (28–166)	115 (NA)	51 (38–64)	62 (NA)	63 (16–182)
Glucose	46 (NA)	43 (22–62)	52 (40–58)	62 (NA)	44 (40–49)	110 (NA)	47 (29–131)
Tests, no. positive/total no. tested							
Conventional CSF pathogen tests							
EV	0/0	17/19	0/9	0/0	0/4	0/0	0/65
HPeV	0/0	0/16	4/8	0/0	0/1	0/0	0/46
HSV	0/0	0/17	0/6	0/0	0/1	0/1	0/24
CMV	0/0	0/0	0/0	0/0	0/0	0/0	0/0
HHV-6	0/0	0/0	0/0	0/0	0/0	0/0	0/0
Other	0/0	0/0	0/0	0/0	0/0	0/0	0/1 (VZV)
CSF culture	0/1	0/21	0/11	0/1	1^*b*^/4	0/1	0/107
Other conventional pathogen tests							
EV/HPeV PCR (blood)	0/0	13/16 (EV)	8/8 (HPeV)	0/0	0/2	0	2/54 (EV)
HSV PCR (blood)	0/0	0/13	0/6	0/0	0/2	0/1	0/46
Urine culture	0/1	0/20	1/9	1/1	0/2	1/1	16/101
Blood culture	0/1	0/21	0/11	0/1	0/2	0/1	2/104
Outcomes, no. (%) of cases							
Admitted	1 (100)	19 (90)	9 (82)	1 (100)	4 (100)	1 (100)	99 (93)
ICU	0	1 (5)	0	0	1 (25)	0	15 (14)
Death	0	0	0	0	0	0	0
Other hospitalization data							
Median hospital LOS in h (range)	142 (NA)	45 (14–157)	43 (26–132)	138 (NA)	53 (33–167)	123 (NA)	70 (16–574)
No. (%) of patients who received:							
Inpatient antibiotics	0	19 (90)	8 (73)	1 (100)	2 (50)	1 (100)	92 (86)
Acyclovir	0	13 (62)	6 (55)	0	1 (25)	0	51 (48)

a Both S. pneumoniae and Cryptococcus were determined to be false-positive results in the parent study (5). For EV and HPeV, one infant was determined to be positive by the FilmArray ME panel. NA, not applicable; NT, not tested.

b One infant with HHV-6 detected by FilmArray ME was diagnosed with bacterial meningitis with Gram-stain positive (Gram-negative rods), culture-negative CSF.

c That is, a WBC count > 14.

Thirty-six infants (25%) had 37 viral pathogens detected from CSF. One infant had two pathogens (EV and HPeV) detected. Of the 37 pathogens, 36 (97%) were detected by the FilmArray ME panel, and 21 (57%) were detected by conventional CSF testing. Of these, 14 infants (39%) had CSF pleocytosis, defined as a CSF white blood cell (WBC) count of >14 (range, 20 to 1,779 not adjusted for red blood cell [RBC] count, which ranged from 1 to 58,130) (13). Twenty-one (58%) infants with a viral pathogen detected had EV, and 11 (31%) had HPeV. Only one infant was found to be CMV positive; this infant was not tested for CMV by conventional methods, did not have CSF pleocytosis, and was diagnosed with a urinary tract infection (UTI). Four infants were positive for HHV-6, none of whom had conventional testing for HHV-6 performed. One of these infants was diagnosed with bacterial meningitis based on a positive Gram stain (Gram-negative rods), with a negative culture; the FilmArray ME panel was also negative for bacterial pathogens. The other three were diagnosed with “viral illness not otherwise specified” and discharged in less than 72 h. No infant had HSV detected in CSF.

Ninety-two percent (33/36) of the infants with a virus detected from the CSF were admitted to the hospital; one was admitted to the ICU. The median LOS for admitted infants was 44 h (range, 14 to 167 h). Three infants (8%) with virus-positive CSF (one CMV, one HHV-6, and one HPeV, all identified by the FilmArray only) had a concomitant bacterial infection diagnosed, including one bacterial meningitis (mentioned above) and two UTIs. Twenty-nine of the infants with a viral detection from CSF (81%) received antibiotics, and 20 (56%) received acyclovir. Fifty percent received both antibiotics and acyclovir.

One hundred seven infants (74%) had no pathogen detected in CSF by either the FilmArray ME panel or conventional tests. The mean CSF WBC count was 4 (range, 0 to 70), but 17 infants (16%) with negative CSF testing had CSF pleocytosis (range, 16 to 70 WBC). Ninety-nine infants (93%) with no CNS pathogen detected were admitted to the hospital, with a median LOS of 70 h (range, 16 to 574). Two had blood PCR results positive for EV. Eighteen (17%) were diagnosed with a bacterial infection, including two infants with bacteremia (one group B streptococcus and one E. coli) and sixteen with UTIs. Most (92/107; 86%) received antibiotics, and almost half (51/107; 48%) received acyclovir.

There were too few infants (*n* = 4) positive for EV by the FilmArray ME panel who did not receive a diagnosis of EV by CSF or blood PCR for statistical comparisons. Table 3, however, shows detailed information for 16 infants with positive CNS EV testing by both conventional methods and the FilmArray ME panel as the change for these infants if the FilmArray ME testing were available would only be in time to a positive EV result. All 16 were admitted, and none had a concomitant bacterial infection. Most (13/16; 81%) had at least one blood viral PCR performed in addition to CSF PCR. Fifteen (94%) received antibiotics, and twelve (75%) received acyclovir. The median lengths of treatment with antibiotics and acyclovir were 2 days and 1 day, respectively. The median length of stay was 47 h (range, 14 to 157 h); 11 infants (69%) were hospitalized for 48 h or less, and 3 (19%) were hospitalized for less than 24 h. The mean turnaround time (TAT) for the CSF EV PCR at the enrolling institutions was 17.3 h and that for CSF HSV PCR was 12.3 h.

**TABLE 3:** Clinical details for infants determined to be positive for EV by both conventional PCR and the FilmArray ME panel

Parameter	Data (*n* = 16)
Demographics	
Median age in days (range)	26 (3–54)
% female	50
No. (%) > 29 days^*a*^	6 (38)
CSF parameters, no. (%) or median (range)	
CSF pleocytosis (WBC count > 14)	12 (75)
WBC count	29 (0–1,779)
RBC count	110 (0–58,130)
% neutrophils	14 (0–92)
Protein	80 (19–525)
Glucose	42 (22–62)
CSF tests performed, no. (%)	
EV PCR	16 (100)
HPeV PCR	13^*a*^ (81)
HSV PCR	15 (94)
CSF culture	16 (100)
Other infectious diagnostics performed, no. (%)	
EV/HPeV PCR (blood)	13 (81)
HSV PCR (blood)	11 (69)
Urine culture	12 (75)
Blood culture	16 (100)
Diagnostics, median turnaround time (h)	
CSF EV/HPeV PCR	17.3
CSF HSV PCR	12.3
Blood EV/HPeV PCR	20
Blood HSV PCR	19.5
CSF culture (final)	96
Blood culture (final)	134
Management, no. (%)	
Admitted	16 (100)
ICU	1 (6)
Antibiotics	15 (94)
Median length of therapy (days)	2
Acyclovir, no. (%)	12 (75)
Median length of therapy (day)	1
Other hospitalization data	
Median hospitalization LOS in h (range)	47 (14–157)
No. (%) of patients:	
Hospitalized ≤24 h	3 (19)
Hospitalized ≤48 h	11 (69)

a EV/HPeV is a combined test at one hospital and accounted for all HPeV tests performed.

## DISCUSSION

Evaluation of young infants for sepsis is common in pediatric practice. LP is often performed, and rapid identification and treatment of infants with serious CNS disease are critical. However, most infants do not have CNS disease, and those who do frequently have a self-limiting viral illness (1). While our study supports a risk of false-positive results with highly sensitive molecular testing (14), a rapid molecular diagnostic such as the FilmArray ME panel could aid in quickly identifying those infants with both serious CNS illness and those at reduced risk. Rapid identification of CNS infection could improve the care of infants undergoing LP and decrease length of hospital stay and antimicrobial exposure for infants with self-limited viral infection detected.

The FilmArray ME panel is a rapid multiplex molecular diagnostic test that can detect and identify 14 common causes of meningoencephalitis (12). The actual run time for the test is about 1 h, and with transport time and results reporting included, an estimate of the time-to-result for the clinician has been made at about 3 h (11). Data from our own institution also show a median time from collection to result of about 3 h (2.75 h [data not shown]). In general, bacterial culture takes 24 to 48 h for results to return. Turnaround time for conventional viral PCR is variable depending on where testing is performed; when testing is local, viral PCR results can return in less than 24 h, but in regions where PCR testing is sent to a central or reference laboratory, the turnaround time can be several days.

In our study, the FilmArray ME panel identified a potential pathogen in 37 of 145 infants (26%), while conventional testing identified a CNS pathogen in 21 (14%). In two cases, the FilmArray ME panel results were likely false positives. This was suggested by secondary PCR testing in the original study (12), and the clinical data from these infants are also supportive. In one case, Streptococcus pneumoniae was detected in an infant with no other signs of CNS infection and a benign hospital course without antibiotic treatment. In the second, Cryptococcus, an uncommon neonatal pathogen (15), was detected. In this case the infant was diagnosed with a bacterial UTI and treated accordingly. No antifungals were prescribed. It is likely that for both of these infants a positive FilmArray ME panel test result would have resulted in further testing and possibly unnecessary treatment. These infants illustrate an issue with highly sensitive PCR-based testing in the setting of a low-incidence infectious process. In the original study there were 22 unconfirmed FilmArray ME panel positive results, including seven unconfirmed S. pneumoniae results and two unconfirmed Cryptococcus results, including those presented here (12). Of note, neither of the two infants described above had CSF pleocytosis, and practitioners may consider delaying the ordering of highly sensitive molecular testing unless initial CSF studies show evidence of infection (14). That said, almost 40% of the EV-positive infants and >90% of the HPeV-positive infants in this study had normal CSF WBC counts, making this a more complex clinical judgment.

Thirty-six (25%) infants in this study had CSF positive for a viral pathogen. EV was the most common virus detected in our cohort, followed by HPeV. Infants with viruses other than EV detected infrequently had CSF pleocytosis, including those with HPeV and HHV-6, a finding consistent with previous reports (16–18). A number of studies have demonstrated that EV detection in young infants presenting with fever decreases the concomitant risk of bacterial infection (1, 4, 6, 19) and that EV-positive infants could possibly be managed conservatively, without antibiotics and with early discharge (1, 13).

Many hospitals have introduced institutional guidelines for the management of infants diagnosed with EV (9, 10), and it has been shown in a number of studies that rapid detection of EV can decrease unnecessary antimicrobials, length of hospital stay, and hospital costs (5, 7, 20). For these reasons, we examined infants with CSF positive for EV by both the FilmArray ME panel and by conventional PCR in more detail, since the only change for these infants if the FilmArray ME panel had been in use clinically would have been the time to EV detection. Management decisions for infants positive for EV clinically could suggest how practice might change with rapid diagnosis. Over half of the infants with EV detected by conventional methods in our study were discharged in 48 h or less, and almost 20% were discharged in less than 24 h. It is likely that infants discharged in under 24 h were discharged at the time that the EV PCR came back positive (median TAT, 17.3 h). Guidelines support early discharge of otherwise stable infants with positive EV testing (1, 10), suggesting that these infants might have been discharged earlier with a more rapid test. Similarly, 75% of infants received acyclovir, but with a median length of therapy of 1 day, suggesting that acyclovir was also discontinued when PCR results returned and might not have been started if EV was known to be positive more quickly (11). Most infants (13/16; 81%) also had blood PCR testing for EV, a relatively costly test that may not have been needed if EV had been rapidly detected in CSF.

HPeV is less well studied, but it has been suggested that with further data these infants may be able to be managed similarly to those who are positive for EV (16, 18). Increasing use of multiplex testing, including HPeV, such as the FilmArray ME panel, may provide these data. In our study, only 1 of 11 infants with HPeV detected in the CSF had a concomitant bacterial infection. HPeV is not regularly tested for at all institutions and/or is often a separate test when ordered. While there is one other U.S. Food and Drug Administration-approved rapid CSF EV test (Xpert EV; Cepheid, Sunnyvale, CA), this test does not include HPeV (21). In the present study, only one of three institutions had guidelines in place recommending routine HPeV testing for young infants undergoing sepsis evaluation, and only infants from this institution were tested clinically. Interestingly, most infants diagnosed by conventional testing with HPeV were PCR positive from blood rather than CSF, despite most (8 of 11) having had CSF testing for HPeV sent and positive FilmArray ME panel results.

Further studies will need to be performed to guide recommendations for evaluation and management of infants in whom herpesviruses other than HSV are detected. In our study, one of the infants with CSF positive by the FilmArray ME panel for HHV-6 was diagnosed with bacterial meningitis based on a positive CSF Gram stain, suggesting that detection of HHV-6 from CSF may not exclude other CNS infections. This has also been shown in other studies (22). HHV-6 is known to be present as a latent infection (23) and can be transmitted from mother to child, both through germ line chromosomal integration and as a congenital infection (24, 25). While we have a small sample set, none of the infants with HHV-6 detected in our study was evaluated for HHV-6 and, except for the infant diagnosed with bacterial meningitis, all were discharged within 72 h without therapy. This is consistent with a previous study by Messacar et al. in which infants with CSF tested clinically for EV were retrospectively evaluated for HHV-6 (16). In that study, there were five infants with HHV-6 detected. No infant with HHV-6 detected had CSF pleocytosis or a documented bacterial coinfection. None were diagnosed clinically or treated for viral infection. In rare cases, serious CNS illness due to HHV-6 has been described; however, this occurs most often in patients with immune compromise (26). Infants presenting with significant illness and detection of HHV-6 from CSF may require further investigation, including an evaluation for abnormal CNS imaging findings, as well as the possibility of congenital HHV-6 or chromosomal integration (24, 25).

Detection of CMV in the CSF of young infants with suspected sepsis is also complex. While detection of CMV may not explain their presentation, it suggests the possibility of congenital CMV infection and the need for additional testing and possibly treatment (27). This is particularly true for infants <30 days of age. Fortunately in this study, incidental detection of CMV in the CSF of young infants was uncommon (12).

No infant in this cohort had CSF positive for HSV, which is consistent with the low incidence of HSV encephalitis in this age group (2, 28). This cohort also did not have any infant positive for HSV by blood PCR, but a word of caution is necessary when discussing the role of the FilmArray ME panel testing in excluding HSV disease in very young infants. Studies of the clinical presentation of HSV in infants <42 days old suggests that many infants have systemic onset, disseminated disease that may or may not include CNS infection (28). Rapidly “ruling out” HSV disease with a test limited to CSF is risky, and we would suggest that all infants clinically suspected of HSV be continued on acyclovir until both CSF and blood HSV PCRs are negative or an alternative diagnosis is found.

Drawing conclusions about potential management changes for infants with negative CSF rapid molecular testing is difficult. While the FilmArray ME panel detects many of the common pathogens of neonatal meningitis (3), including group B streptococcus and E. coli K1, only six bacterial pathogens are represented, and early neonatal pathogens such as other E. coli serotypes and Citrobacter spp. are not included. Thus, in infants with a high suspicion for meningitis, antibiotics should not be stopped based on a negative test (14, 29). Seventeen percent of the infants in our study with negative FilmArray ME panel testing had a non-CNS bacterial infection (14% with UTI, 2% with bacteremia), demonstrating the need for continued evaluation and antibiotic therapy for children with negative CSF PCR results. Caution regarding the evaluation and management of HSV in infants based solely on negative CSF testing has been discussed above.

Our study has several limitations. While this is the largest study to date examining the use of multiplex molecular testing for CNS pathogens in young infants, there were a limited number of positive detections, and sample sizes were too small for statistical comparisons. Most infants with pathogens detected were positive for viruses, and we could not evaluate the impact of molecular testing on the detection and management of bacterial meningitis. No infant had HSV detected, and the impact on HSV infection also could not be evaluated. Our study was performed retrospectively, and the results of FilmArray ME panel testing were not shared with providers. Thus, we can only postulate how management may have changed if results were available. Granular data on hospital costs were not available such that conclusions could be made regarding the potential effect of rapid testing; however, this is a critical measure of the impact on care.

### Conclusions.

Young infants are often evaluated for sepsis and LP is frequently performed. The FilmArray ME panel can rapidly detect a number of the most common pathogens causing meningitis/encephalitis in young infants and may play a role in their evaluation. Clinical judgment should drive both the choice to use this test and interpretation of the results with regard to further management. The results of rapid multiplex PCR testing may be used to guide further work up and antimicrobial therapy, possibly resulting in decreased LOS and antimicrobial exposure for infants with viruses such as EV that are rapidly detected.
